# Prospects for progress on health inequalities in England in the post-primary care trust era: professional views on challenges, risks and opportunities

**DOI:** 10.1186/1471-2458-13-274

**Published:** 2013-03-26

**Authors:** Daniel Turner, Sarah Salway, Ghazala Mir, George TH Ellison, John Skinner, Lynne Carter, Bushara Bostan

**Affiliations:** 1 Centre for Health and Social Care Research, Sheffield Hallam University, 32 Collegiate Crescent, Sheffield, S10 2BP, UK; 2 Leeds Institute of Health Sciences, University of Leeds, Charles Thackrah Building,101 Clarendon Road, Leeds, LS2 9LJ, UK; 3 Leeds Institute of Genetics, Health and Therapeutics, University of Leeds, 8.001 Worsley Building, Leeds, LS2 9JT, UK; 4 NHS Sheffield, Town Hall, Pinstone Street, Sheffield, S1 2HH, UK; 5 NHS Airedale, Bradford and Leeds, Douglas Mill, Bowling Old Lane, Bradford, BD5 7JR, UK; 6 NHS Leeds, Reginald Centre, 263 Chapeltown Road, Leeds, LS7 3EX, UK

**Keywords:** Commissioning, Health inequalities, NHS, General practitioners, CCG, Restructuring

## Abstract

**Background:**

Addressing health inequalities remains a prominent policy objective of the current UK government, but current NHS reforms involve a significant shift in roles and responsibilities. Clinicians are now placed at the heart of healthcare commissioning through which significant inequalities in access, uptake and impact of healthcare services must be addressed. Questions arise as to whether these new arrangements will help or hinder progress on health inequalities. This paper explores the perspectives of experienced healthcare professionals working within the commissioning arena; many of whom are likely to remain key actors in this unfolding scenario.

**Methods:**

Semi-structured interviews were conducted with 42 professionals involved with health and social care commissioning at national and local levels. These included representatives from the Department of Health, Primary Care Trusts, Strategic Health Authorities, Local Authorities, and third sector organisations.

**Results:**

In general, respondents lamented the lack of progress on health inequalities during the PCT commissioning era, where strong policy had not resulted in measurable improvements. However, there was concern that GP-led commissioning will fare little better, particularly in a time of reduced spending. Specific concerns centred on: reduced commitment to a health inequalities agenda; inadequate skills and loss of expertise; and weakened partnership working and engagement. There were more mixed opinions as to whether GP commissioners would be better able than their predecessors to challenge large provider trusts and shift spend towards prevention and early intervention, and whether GPs’ clinical experience would support commissioning action on inequalities. Though largely pessimistic, respondents highlighted some opportunities, including the potential for greater accountability of healthcare commissioners to the public and more influential needs assessments via emergent Health & Wellbeing Boards.

**Conclusions:**

There is doubt about the ability of GP commissioners to take clearer action on health inequalities than PCTs have historically achieved. Key actors expect the contribution from commissioning to address health inequalities to become even more piecemeal in the new arrangements, as it will be dependent upon the interest and agency of particular individuals within the new commissioning groups to engage and influence a wider range of stakeholders.

## Background

The documentation of inequalities in health has a long history in England [1,2]; and the importance of tackling such inequality has received sustained policy attention for the past 15 years. Tony Blair’s Labour Government signalled its intention to take health inequalities seriously by commissioning an independent enquiry led by Donald Acheson in 1998 and a subsequent cross-cutting review in 2002 [3,4]. In 2001, explicit targets were set for reducing health inequalities by 2010, followed soon afterwards by a Programme for Action [5] and the Health Inequalities National Support Team (HINST). The current Conservative-led Coalition Government has indicated that it will retain this focus on health inequalities, and has confirmed its intention to act on the findings of a wide-ranging review commissioned by the previous Labour government - *Fair Society, Healthy Lives*[6]. Thus, policy commitment to addressing health inequalities now appears to be an accepted part of the UK political mainstream.

Tackling health inequalities has been portrayed as a collective effort, bridging local and national government responsibilities [5]. Local Strategic Partnerships, which were established in 2000, are intended to coordinate action across housing, employment, environment and other wider determinants of health. Likewise, Joint Strategic Needs Assessments (JSNAs), which were introduced in 2007, are intended to be the vehicle through which local partners should systematically identify unmet local needs and priorities for intervention [7].

Since their development from 2001, Primary Care Trusts (PCTs) had responsibility for planning and funding the majority of health services, in a process known as commissioning [8]. There have now been two decades of experience in commissioning health services in this way, with the World Class Commissioning agenda from 2007 increasing the professional learning in PCTs on the commissioning process, and shifting practice to a coherent cycle of commissioning. During this whole period, a central role for the NHS in addressing health inequalities was clearly articulated; in particular PCTs were expected to highlight action on inequalities in the way the NHS delivers its services, ensuring that service redesign narrows health inequalities [9]. There was a concomitant expectation that PCTs led on this agenda locally, engaging partners to ensure that services supported health improvement and narrowed inequalities. Strategic Health Authorities (SHAs) were charged with supporting PCTs, providing a locus for planning, performance management, and developing the appropriate skills and knowledge, including around addressing health inequalities [10].

Nonetheless, despite these structures and the apparent political commitment, progress has been disappointing. Independent analyses show that inequalities in life expectancy actually increased during this period, [11,12] and such trends led a recent King’s Fund review of the NHS to identify the lack of progress in reducing health inequalities as the most significant health policy failure of the last decade [13].

A variety of lines of critique have been advanced to account for this disappointing progress. The most important of these can be broadly grouped into the following four areas of weakness: (i) lack of serious attention to social and economic inequality, i.e. the structural determinants of inequalities in health; (ii) failure to shift resources from secondary and tertiary services to high quality prevention, early diagnosis and treatment in primary care; (iii) lack of performance management; and (iv) persistence of a weak evidence base.

On the first of these, commentators have argued that progress is hampered by a lack of interventions to reduce the social and structural inequalities that underline differences in health. Instead, national government has largely promoted interventions that target individual behaviour and health-damaging ‘lifestyle choices’ [2,14], as well as some that take a social integrationist approach, seeking to strengthen community resources, resilience and social capital, often with limited success [15,16].

On the second, attention has been drawn to the failure of PCTs to shift NHS expenditure away from secondary services and towards prevention, early detection and treatment. Critiques of PCT commissioning have highlighted limited impact in service change [8], especially in ensuring that providers, particularly large hospital trusts, reshape historical service patterns [17], or prioritise resource allocation to reduce inequalities [18]. Even specific initiatives intended to eliminate variations in access and quality of primary health care have had mixed success. In 2010, the Committee of Public Accounts openly criticised the Department of Health for failing to ensure that primary care provision and preventive interventions such as smoking cessation were rolled out to include deprived areas [19].

Similarly, PCTs have struggled to get large provider trusts and general practitioners to look beyond the patients they see and take responsibility for tackling inequalities in the health of the wider population. Since 2003, the Quality Outcomes Framework (QOF), has involved GP practices being financially rewarded for the proportion of eligible patients for whom specific targets (measured by clinical activity indicators) are achieved. Yet, while overall performance in achieving these targets has improved, many of these targets can be met without tacking inequalities, or by excluding difficult cases, providing little incentive for practices to undertake primary prevention or public health interventions [20].

The third line of critique notes that the intended performance management of NHS organisations against health inequalities targets has simply failed to materialise, indicated by the failure to meet Public Service Agreement targets to reduce health inequalities by 10% by 2010 [8]. Exworthy et al. [21] have noted that since responsibility for tackling inequalities is divided among several agencies, it is difficult to determine each agency’s contribution, and incentives for taking action therefore lack specificity, relevance and leverage [22]. Where these incentives do gain purchase, they are predominantly based on financial and process-related service targets that continue to dominate the NHS, despite rhetoric to the contrary [23]. Consequently it is not surprising that inequalities work is tacitly acknowledged to be less important than efficient service delivery, and staff are neither rewarded nor sanctioned for their performance on the health inequalities agenda [21]. Indeed, there is evidence of a substantial need for greater leadership and workforce capacity within the NHS to engage with the health inequalities agenda [24].

Finally, it has been argued that the evidence base on tackling health inequalities remains weak, and that a continuing lack of rigorous evaluation of interventions intended to address inequality means that policy-makers and commissioners have little to inform their allocation of resources [21,25]. Some PCTs and SHAs have managed to offer a stronger strategic focus on health inequalities, yet many promising pilot interventions have not been well evaluated or promoted, restricting the scaling up that is required to effect health improvement at the population level. Furthermore, Health Equity Impact Assessments, though advocated as early as the 1998 Acheson Report, have tended to lack detail, so that the unintended deleterious effects of some public policies on health inequalities have been unanticipated and unmitigated.

**Figure 1:**
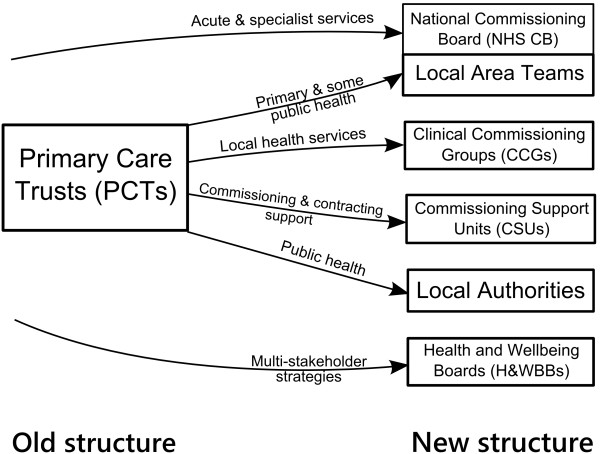
Summary of changes to NHS commissioning organisations.

While reducing health inequalities remains a clear priority for the current government, the structures within which commissioning work is to be achieved are undergoing radical change, with the role of PCTs being abolished, and responsibility for commissioning many local health services moving to groups led by General Practitioners (GPs), structured as “Clinical Commissioning Groups” (CCGs). These groups will also include clinical nurse, hospital doctor and lay representation. PCTs will evolve into Commissioning Support Units, offering assistance on contracting and commissioning to CCGs. A National Health Services Commissioning Board (NHSCB) will have an overarching role in commissioning primary care and specialist health services through a network of regional teams. The restructuring also involves public health functions being relocated within Local Authorities, and adjustments to the regional and national supporting architecture including locally based Health and Wellbeing Boards which will bring together local stakeholders in health from the NHS, CCGs, Local Authorities and even third sector and academic representation. HealthWatch groups will replace Local Involvement Networks (LINks) to provide patient and lay scrutiny and represent patient voice as consumers [26]. These developments were still in flux during the research project and the publication of this article, but Figure 1 gives a summary.

Not surprisingly, there has already been heated discussion on what the changes may mean for future progress on the health inequalities agenda. Pollock et al. [27] argues that the watering down of the Secretary of State’s duty to provide a comprehensive health service, CCGs’ apparent responsibility for registered patients rather than geographical populations, and the relocation of public health without clear specification of the services Local Authorities are duty-bound to provide, could all significantly increase inequalities in access to healthcare, and the provision and uptake of health services. The Public Health Outcomes Framework does state that ‘outside the clinical arena’ responsibility for reducing health inequalities will lie with Local Authorities, and that health and wellbeing boards will drive integration of action to improve health ([28], p6). However, the King’s Fund has expressed doubts that the duties on CCGs and Local Authorities, and the powers of the Health & Wellbeing Boards are sufficient to ensure that priorities are aligned and population-level inequalities in health are given adequate attention. There are also concerns that CCGs adequately understand the scale and value of local voluntary and community organisations and how to work with them to address inequalities [29]. Meanwhile, Iacobucci [30] has noted that the formation of CCGs around groups of GP practices appears to be creating a greater clustering of deprivation in some commissioning localities, raising the question of how this may affect action on inequality especially as it is proposed that funding for health services is allocated based only on the proportion of older people in a region [31]. Finally, there is a concern that knowledge and expertise in tackling health inequalities in specific populations, particularly relating to community engagement, will be lost during the transition [32].

Clearly, there is a great deal of unease and conjecture. Nevertheless, the impact of government reforms will depend not so much on the written policies and plans but rather on how these are operationalised. Since implementation lies in the hands of regional and local actors, their perspectives and experience can potentially highlight future risks and opportunities. The aim of this paper is therefore to inform current debates by reporting findings from a series of in-depth interviews conducted with a range of experienced professionals working in varied roles within the health and social care commissioning arena. While commentary on the implications of current reforms for future progress can only ever be speculative, the respondents interviewed are experienced, well-informed and key actors in this unfolding scenario.

## Methods

The findings reported here come from analyses of the first phase of data collection conducted as part of a large, NIHR/HS&DR-funded project investigating the use of evidence in healthcare commissioning for multi-ethnic populations (http://www.eeic.org.uk). Whilst the focus of the study was primarily on ethnic health inequalities this was explored in the context of wider commissioning activity and work to address health inequalities in general.

Ethical approval was obtained from NRES East Midlands, and semi-structured interviews were conducted between October 2010 and June 2011 with experienced professionals working in varied roles within the health and social care commissioning arena. Expert, in-depth interviews have been found to be well-suited to gaining insights into the structure and functioning of complex environments, including healthcare policy making [33]. Semi-structured interviews were used to provide a focus and structure but still allow for the interviewer to probe further, and for the interviewee to add extra detail where appropriate [34]. These interviews were designed to generate insights into the key characteristics of healthcare commissioning work, from a broad range of perspectives, and particularly into the factors that facilitate or hamper progress towards reduced inequalities.

### Sampling

A purposive approach that combined elements of maximum variation and critical case sampling was used to identify suitable respondents [35] assisted by snowballing sampling [36] beginning with local and national contacts known to members of the research partnership. Recognising the broad range of actors and organisations that shape commissioning work at both national and local levels, we identified two sets of respondents offering varied perspectives and rich descriptions of the commissioning arena. At the national-level, these comprised of individuals with extensive experience and interest in the health inequalities agenda as well as significant engagement with relevant national-level policy formulation. At the local-level, respondents were identified across three case study sites in the north of England as people having significant experience of healthcare commissioning structures and processes, even if they did not necessarily have a particular interest in health inequalities work.

### Data generation

An interview topic guide was developed, piloted and refined [37] to provide a loose structure for the interviews covering: professional background and experiences; commissioning structures, networks and processes; commissioning impact; role of evidence and knowledge in commissioning; barriers and opportunities for improved commissioning to address inequalities; and the implications of new commissioning arrangements for such work. Researchers also completed a reflection and summary template after each interview to capture their impressions on the content and process of the discussion.

Data was generated through face-to-face and telephone interviews at a time and place convenient to respondents. It was envisaged that around 40–50 detailed interviews would be needed to capture the full range of stakeholder perspectives relevant to commissioning and health inequalities.

### Analysis

Data analysis was informed by a theoretical perspective which viewed healthcare policy making as a process of dialogue and argument within which power relationships are key and policy-makers piece together information from diverse sources to make decisions within the context of multiple competing drivers [38-41]. This theoretical stance enabled us to view commissioning activities as dynamic interactions between individual agency, organisational rules, structures and processes, and the wider healthcare setting with its current restructuring agenda, all situated within the broader socio-political context. This also draws on broader theoretical perspectives that view policy making as a process of collective interaction between diverse stake-holders in which both the identification and responses to problems are socially situated and constructed [42,43]. As such respondent narratives were seen both as a representation of personal attitudes and perspectives held by a key set of actors, but also as a window onto the complex processes operating within the commissioning arena.

At an operational level, interviews were recorded and transcribed verbatim, with researchers also preparing reflexive field notes on each interview soon after their completion. Following an initial reading of interview transcripts, a coding scheme was developed, piloted and refined iteratively by all team members; combining both an inductive data-driven and a theory-driven approach. Microsoft Excel was used to organise the derived codes (around 120 grouped into 10 broader themes) into a practicable framework that was then used to index the interview material [44]. The initial coding of transcripts was undertaken in most cases by the team member who conducted the interview, and around 20% of transcripts were then checked by a second member of the team to clarify areas of inconsistency. The framework made it possible to establish which themes were present in a particular interview and examine each thematic code across all interviews, thereby maintaining the context of each data extract while combining these across interviews to explore commonalities and contrasts. Finally, team members engaged in analysis workshops to discuss, challenge and refine interpretations and claims.

One of the broader themes captured respondents’ opinions on the future of health commissioning and the implications of current re-structuring for work to reduce health inequalities, from which much of the data presented in this paper is drawn.

## Results and discussion

Response rates were high with only two potential participants declining to take part, both of whom cited work pressures. A total of 18 national expert interviews were conducted with individuals who held, or had recently held, senior roles within Strategic Health Authorities, the Department of Health, Public Health Observatories, Local Government umbrella organisations, GP bodies, and third sector organisations. In addition, 24 local expert interviews were conducted with people working within public health and other directorates of PCTs, Local Authority commissioners, GPs who were assuming commissioning roles, and staff members of third sector organisations with a focus on health inequalities (see Table 1). Many respondents at both national and local levels had experience of working in more than one relevant organisational setting, and some had multiple roles: their primary job title is reflected in the rough categories in Table 1.

**Table 1:** Primary role and organisation of interviewees SHAPE

**Role**	**N of interviewees**
PCT commissioners and managers	11
Department of health / strategic health authority	4
GP and CCG members	5
Third sector managers	9
Academic	2
Public health commissioners and observatory managers	6
Local authority commissioners	7
**Total**	**44**

The majority of interviews were held face-to-face, with three being conducted by telephone at the request of respondents. Respondents were asked to present their own personal experiences and reflections, in addition to official organisational policy. Researchers’ reflections suggested that in most cases respondents were willing to express critical as well as positive perspectives on their experience of commissioning processes and activities. The following section first presents a brief overview of participants’ reflections on progress towards tackling health inequalities during the PCT era, followed by their views on future prospects. These findings are organised into three broad themes: opportunities and risks for the future; skills and competencies; and partnerships and engagement. Each section begins by highlighting the positive opportunities that respondents noted, and then considers any challenges and risks that they foresaw.

### Past progress on commissioning to reduce health inequalities

Respondents generally felt that there had been pockets of good practice in commissioning to reduce health inequalities. These tended to occur where a pressing need had been identified, or where services and contracts were smaller and less dominated by existing block contracts, and therefore easier to change. When asked if commissioning had a significant influence on services, one respondent replied:

*“It depends on whether you are commissioning from a blank sheet or commissioning an existing service. They’re quite different scenarios… a lot of services which were never specified, chugged along… And then there have been new services where they have decided to take the money out and try it another way through open tender”* - Public Health Consultant

A common theme was that it is easier to inject attention to health inequalities when the commissioning task involves a new service, rather than the redesign of an existing one, and when new funding was available, this had provided opportunities to deliver new services and new providers through open tender.

Where circumstances allowed, a good number of respondents still felt that commissioning can be an important tool for service improvement to address inequalities.

*“The real lever for change lies within commissioning”* - PCT manager

*“We are pretty effective at understanding what needs to change about services, redesigning and being able to commission”* - Senior PCT manager

However, with another NHS restructuring being enacted, there was widespread recognition of the risk to this progress:

*“Every time we’ve had a change of gear or direction in terms of the commissioning agenda, the sophistication that had been reached gets lost; everybody goes back to the beginning” -* Public Health Observatory manager

There was a general feeling that commissioning had, over time, improved and that PCTs had become more skilled over the last decade at enacting commissioning, including around inequalities. Many PCT commissioners and other respondents expressed concern that this progress could be wasted as a result of reorganisation:

*“I’m really nervous about it and I think good practice might be lost” -* Third sector manager

Most respondents agreed that the PCT commissioning era had failed to deliver significant progress towards reduced health inequalities, and many reiterated the shortcomings documented in the published literature already highlighted above.

Several respondents highlighted the poor track record of PCTs in shifting resources out of secondary care and into the types of primary care and public health interventions felt to be capable of achieving a significant impact on health inequalities. Interviewees referred to the predominance of “transactional” commissioning work (that is the maintenance of existing contracts) rather than “transformational” action that they felt was required to make any real progress. As such, respondents usually felt that the bulk of commissioning activity was tied up in the momentum of historical contracts, with little resource left for innovative redesign:

“*The momentum of the historical contracts has meant… a lot of what the commissioners are doing is almost working at the margins; where you’ve got extra resources you can have a discussion about how they’re best deployed, obviously most of the resources is in a basic set of core contracts*” - Public Health Observatory manager

Respondents’ views also highlighted a perception that responsibility for the health inequalities agenda was seen primarily as a function of Public Health roles rather than part-and-parcel of core healthcare commissioning work, even where PCTs had adopted explicit strategic priorities relating to inequalities.

“*Meeting health inequalities is part of the bread and butter of Public Health*” - Public Health Observatory manager

“*In terms of bringing evidence to the table around need… that is more of a Public Health role*” - Public Health commissioner

While respondents recognised that many organisations had policy statements and process documents reflecting an apparent commitment to addressing inequalities, they felt these did little to influence action. Some respondents also accused PCTs of tokenism in relation to health inequalities, particularly when the focus was on inequalities experienced by ‘protected groups’ such as minority ethnic people. One respondent noted how PCTs often had one or two project examples that would be widely cited as evidence of their work on inequalities; what he referred to as their ‘get-out-of-jail-free-card’ [DH project manager].

*“We do these sort of cursory tick box exercises, which frankly are not helpful, because we do them, then we put them in a drawer and forget all about them*” - PCT commissioner

“*There is a part of the world of equality and diversity, equality impact assessments and some of the hoops which you have to be seen to be going through to demonstrate that you are competent and aware… for me that feels slightly tokenistic*” - PCT commissioner

Many respondents also highlighted the failure of Joint Strategic Needs Assessments - an exercise that was supposed to be conducted between PCTs and Local Authorities to identify health and wellbeing needs, review provision and set priorities for investment - to really have an influence on mainstream commissioning action in relation to inequalities. While there was awareness of the intended function of these documents/exercises, there was general agreement that they were not used systematically and had little impact on commissioning practice.

“*Whether people take full ownership of the Joint Strategic Needs Assessment, and whether every single commissioner in this district uses it - I would question that*” - PCT Public Health commissioner

More generally, respondents felt that the use of data and evidence about local health needs and inequalities was very variable in commissioning, and that there was a particular lack of data availability and use relating to specific axes of inequality:

“*Even the evidence that we have isn’t being used in order to inform how we commission, how we target, how we provide particular services*” - Public Health Observatory manager

“*The health equity audit has been very much focused on either geography, and even that’s a proxy for social class really… and some of the other dimensions like ethnicity have been underplayed*” - Public Health Observatory manager

### Opportunities, challenges and risks for the future

In terms of the prospects for addressing inequalities in the future, some respondents felt that local levers, in particular the Joint Strategic Needs Assessment and Health & Wellbeing Strategies, could significantly shape CCG commissioning actions in the new era:

“*The Health & Wellbeing Board are a key lever for some of this work as well, and the JSNA’s are a critical tool. Then Local Authorities will also have other tools in terms of they have done quite a lot of work on different wards and the needs of those wards - they’ve done lots of work on different interest groups, they’ve had their own community and neighbourhood strategies and all sorts*” - SHA Equality and Diversity Lead

Indeed, there was a degree of optimism that the new local structures and processes, especially those based in Local Authorities, could improve accountability to the general public and therefore increase attention to inequalities:

“*Local government… their track record on ensuring equality in things like housing, you know, and education and so forth, they’ve been much less squeamish about collecting data and using it*” - Third sector manager

Nonetheless, many respondents expressed doubts about the likely representation of community interests on the emergent Health & Wellbeing Boards and whether in practice they would have the teeth to hold CCGs to account.

“*What I don’t know is how much the health and wellbeing boards and their local strategies will be able to influence the GP commissioning groups, you know, who may be focused on the patients that they see most often*” - Public Health Observatory manager

#### Future commitment to the health inequalities agenda

Across the board respondents felt there was a real danger that recognition and commitment to health inequalities would be weakened in the new arrangements. Three key concerns were highlighted: (i) weakened directives from central government; (ii) clinical commissioners’ lack of engagement with a population perspective; and (iii) reduced public health input into health services commissioning.

While some respondents felt that CCG-led commissioning could be positive in localities where GPs had been able to see the impact of inequalities in their local population (and were therefore able to address this directly), even where GPs were already committed to this agenda, respondents felt that the current emphasis on localism and the “hands off” approach by national government would result in piecemeal attention to health inequalities that relied on enthusiastic individuals to stimulate activity:

“*Some of the consortia that we’re dealing with, I think will take this [health inequalities] very seriously, and probably embed it in their work, but I think that’s more spontaneous, from their own drive*” - Third sector manager

Respondents worried that weakened directives from central government on the importance of addressing health inequalities would mean that other priorities - particularly financial probity and efficiency - would likely dominate CCG work.

“*I fear that we may have a period of about eighteen months where that [health inequalities work] is put on hold because there is so much else for folk to juggle with*” - PCT information manager

“*If their main focus is on financial balance, a lot of the work that they’ll be doing will be precisely that, commissioning to try and manage the finances, and will not be commissioning to move the community in a direction for better public health necessarily*” - General Practitioner

Across local and national interviews, respondents felt that GPs did not, by and large, have an awareness of inequalities in service access, experience and outcomes. Respondents speculated that GP commissioners would tend to draw on their experiences in the consultation room, and that this would undermine attention to under-served groups.

“*GPs look at the patient in front of them or on their list, and not at the people that don’t access them, or are not coming through the door*” - Third sector manager

“*Most of general practice is not engaged in the inequalities agenda at all… Most GPs don’t have skills and interest in this area*” - Third sector manager

“*What they’re being given is a huge task that they didn’t necessarily ask for, nor are they necessarily equipped to undertake because most of them aren’t involved at a strategic level; they’re sitting every day in their surgeries practising medicine*” - Department of Health manager

Thus, while GPs were felt to have strong clinical skills and experience with patient care pathways at an individual level, respondents felt that, as CCG members, GPs might have a poor understanding of the importance of taking a population perspective on inequalities.

“*There are lots of strengths to [CCG commissioning]. I hope that it is much more people-centred, but the people [i.e. service users] that it will be centred on are those who are engaged with primary care practices, and there is a risk that those people who have greatest difficulty in engaging will be the ones who miss out*” - PCT information manager

“*[GPs are] too close to the coal face, so that they lack the bird’s-eye vision of the whole picture*” - General Practitioner

Some respondents worried that the move of public health away from the healthcare commissioning function to Local Authorities would further dilute consideration of action on health inequalities by the new CCGs, particularly since they might be less likely to draw on public health intelligence data.

“*I think the PCTs, warts and all, they did have a sort of area based view - ‘This the area that I’m responsible for, this population’. They had the overview across all health care… I think they covered public health inequalities and NHS care… I think this whole divide between public health and… [healthcare commissioning], it’s divisive, it mitigates against…*” - Third sector manager

Respondents also worried about how CCGs could be encouraged to commit resources to prevention and health promotion, without such work being labelled as public health and therefore beyond their remit.

#### Skills and competencies to deliver on health inequalities

Respondents were worried that CCG commissioners would overlook the need for effective community engagement and were not generally well equipped to deal with the politics and emotions that are inevitably faced in the commissioning role, suggesting that they would need “a lot of hand holding” - PCT Public Health commissioner.

There were also general concerns about the loss of good practice and expertise in health inequalities work, particularly since some of the key skills, such as patient and public involvement (PPI) and equality and diversity (E&D), were seen to have been shed from PCTs early on during re-structuring in many places. These skills and the networks that had been built up with communities by engagement and equality officers would be lost from many commissioning support organisations, and CCGs would have to start again from scratch, mirroring the loss of connectivity and effective working that occurred as a result of earlier reorganisations.

Meanwhile, respondents felt that the small size of CCGs would inevitably mean that these would include a narrower range of skills than currently embedded in PCTs and that the likely reliance on ad hoc, consultancy inputs was undesirable:

“*No doubt many [CCGs] will subcontract, they may contract to private companies. It depends on what skills and interest reside there, what focus, what priority those contractors will put on addressing inequalities… It’s very uncertain I think*” - Third sector manager

In addition to voicing concerns regarding the steep learning curve facing the new commissioners, several respondents also bemoaned the loss of national support structures and regional networks that had in the past been important mechanisms for enhancing skills, competence and knowledge on how to address health inequalities.

“*The challenge for us going forward is to keep some of that network going, because the regional structures have gone. And also some of their national structures have gone. So if we change tack slightly, the National Mental Health Development Unit, if I had wanted information on, specific services or evidence of effectiveness around ethnicity, it might have been one of the places I started looking for evidence, or even picking up the phone. Now that was disbanded*” - Third sector manager

#### Partnerships, influence and engagement

Closely related to worries about CCG skills mentioned above, it was notable that many respondents explicitly discussed the requirement for the new commissioners to be able to work in partnership, engage and exert the necessary influence to bring about action on inequalities.

Alongside the significance attached to professional networks, most respondents highlighted the importance of a broader spectrum of partnerships and engagement. On the positive side, some respondents felt that CCGs would be able to make use of their peer networks and clinical knowledge to bring about improvements in primary care access and quality, and would exploit their shared background to gain access, influence and cooperation where PCT managers had often struggled in the past.

“*I think if you come from primary care, so again it’s almost like coming from within, and you’re trying to sell the package back to your peers. There’s much more of a buy-in there, really*” - General Practitioner

However, opinions were mixed as to whether such influence would extend to the hospital trusts, the major consumer of resources, with some respondents feeling the GP commissioners would be completely out of their depth in negotiations concerning the hundreds of contracts PCTs currently held,

“*I keep describing it as is an ecosystem and unless you understand the whole or the chunks of it you’ll miss out*” - Public Health manager

Several respondents, who perhaps saw GPs as obstacles to health inequalities work in the past, felt it was a positive development to have GPs properly engaged in the commissioning process, and felt that this would help to ensure greater cooperation in working towards addressing inequalities, since primary care has such a key role to play.

“*It’s a very powerful opportunity. Now in addition to being the main providers, they’re the commissioners, so it’s all come together*” - Third sector manager

Similarly, some local third sector respondents saw potential benefits to future working with GPs as commissioners and welcomed the possibility that this new role might connect them more effectively with the communities they serve. There were also signs across all respondent groups that at least some GPs are aware of their need to learn from, and engage with, wider partners, including members of local communities, if they are to undertake their new role effectively.

Some respondents also highlighted positive opportunities for new partnership work in pursuit of reduced health inequalities. For instance, the potential for greater integration of knowledge and data on local communities, stronger JSNAs and better understanding of needs, was noted with the move of public health to Local Authorities. In addition, new structures, particularly the HWBB, created the possibility of new opportunities for representation.

However, despite these positive views, the general feeling was that restructuring presented significant risks to partnership working. Several respondents emphasised that partnerships between organisations are fundamentally based on personal relationships and that these take time to establish.

“*If you’ve got that longer standing relationship you can also have a more full and frank discussion and not skirt around some of the issues and take forever and a day… it takes a long time doesn’t it [to develop those relationships] and time is money*” - Public Health manager

Several respondents also noted that GPs were, by-and-large, not used to working in partnership or taking on the statutory duties that CCGs will be charged with. Respondents particularly noted their lack of understanding of how Local Authorities operate and the consequent risk that their links with public health would not be functional in terms of balancing a range of priorities.

“*People recall fund-holding as something where they had a lot of autonomy, a lot of control. Commissioning has moved on in leaps and bounds in different ways since then, and it’s a very different variable beast, where you’re having to deliver national things which you may not agree with, but you’re the conduit for it, and at the same time you’re responding to your local community, and you’re trying to balance the two, because sometimes they conflict*” - General Practitioner

Both GPs and people working in PCTs noted that relationships between the two groups were not always cordial. Caution was also expressed by both GPs and other respondents that some GPs would take the opportunity for independence from the PCTs to commission in completely different ways, and in so doing ignore years of experience and success in many areas.

“*There’s a lot of bad blood between PCTs and GPs, for historical reasons, a lot of tension and friction over the years, and some GPs will tell you ‘oh, we don’t want anything to do with PCTs ever again*” - General Practitioner

“*Lots of relationships that are built up are disrupted, now sometimes that’s an opening for new relationships and people that haven’t had a seat at the table, but often you can throw the baby out with the bathwater, and I’m a bit concerned about that*” - Third sector manager

Some respondents in PCTs expressed a more general hostility to the notion of GPs taking over commissioning. “GPs have to be paid to do anything!” was an extremely common refrain from respondents who saw themselves as champions for inequalities issues, suggesting that they did not view GPs as obvious allies and that GPs had considerable work to do to establish a positive image in the eyes of these actors.

Several respondents expressed concerns that CCGs would have to start a lot of community engagement work from scratch, and develop meaningful relationships with key communities. Engagement was seen by many participants to be important not just for understanding population needs, but also in commissioning services that effectively meet those needs.

”*Good commissioners get out there and talk to parents, carers, families; they have many mechanisms to understand how it feels to be receiving services*” - PCT healthcare commissioner

Other respondents expressed doubts as to whether GP commissioners would even recognise the importance of finding ways to engage with the wider population, again underscoring the need for CCGs to develop partnership approaches with Local Authorities, who often had greater strengths in this area.

## Conclusions

Our findings highlight general agreement with the critique of PCT progress on health inequalities outlined in the introduction to this paper and with the need for improvement on the PCT commissioning era. Historically, health inequalities work was generally perceived as the business of public health staff and assessments of unmet need had largely failed to influence mainstream commissioning action. The momentum of large contracts with provider trusts left little room for commissioning to specify inequality objectives in contracts or to shift spend towards prevention and earlier intervention. Respondents felt that there had been pockets of good practice, and that skills were developing, but nevertheless painted a picture of limited organisational engagement and low priority accorded to tackling health inequalities.

Notwithstanding this past performance, our respondents were generally doubtful about clinical commissioning taking any clearer action on health inequalities in the near future. They expressed concern, noted elsewhere [45] that many GP commissioners lack the necessary skills and experience, and that their immediate concern would be to get to grips with the transactional aspects of commissioning with little time for transformational work. Further, the demise of PCTs was felt to risk the loss of expertise and whatever good practice there was in this area – repeating respondents’ experiences of past restructuring exercises. Participants also perceived a significant weakening of national structures and leadership in support of health inequalities work, meaning that CCGs might not be prompted to undertake action if they were not already committed to this agenda.

There was, in addition, a feeling that GPs would need to shift their worldview in order to recognise their new responsibilities for population health and appreciate the needs of disadvantaged groups and non-service users. Many respondents’ comments suggested that GP commissioners will have much to do to win the trust and respect of colleagues who have a commitment to health inequalities work. Linked to these negative perceptions of GPs, a worrying theme emerging from several of the interviews was the potential for historically poor relationships to limit co-operation between CCGs and other bodies. This is in addition to existing speculation that GPs will have low levels of engagement and poor relationships with LA colleagues and third sector organisations. The cumulative effect of these potential limitations confirms suggestions that clinical commissioning may have little impact beyond primary care [46] and indicate that HWBBs may struggle to get deep cross-sectional commitment to inequalities work.

Despite these generally pessimistic views, it was clear from both national and local expert interviews that there is substantial variation in the quality of collaborative relationships, and that some PCT managers, LA commissioners and GPs had cultivated very positive working relationships in the past. Some respondents’ narratives also suggested a number of potential opportunities for action on health inequalities presented by the emerging structures, roles and responsibilities. In particular, there were hopes for greater accountability of commissioners to local communities via the HWBBs as well as stronger JSNAs and more coordinated work to address wider social and economic determinants resulting from public health’s move to Local Authorities. Further, several respondents were optimistic that, even though it was likely to be patchy, at least some of the CCGs would take inequalities seriously, and that commissioning GPs’ professional identities, clinical expertise and awareness of local needs would enable them to bring about positive change despite a weaker national policy context. This observation tallies with other analyses that emphasise the importance of local contextual factors and the individual agency of policy makers in mediating the effect of policy interventions [21].

Elsewhere though, the tone of many interviews was pessimistic. This may simply reflect widespread disillusionment amongst the individuals interviewed during a period of unprecedented insecurity and uncertainty. Some respondents are also likely to be jaded veterans of repeated reorganisations and associated false starts across a range of health service policies over the past decade. Their prognosis of GP-led commissioning’s capability for addressing inequalities in health should therefore be understood in the context of past failures (exacerbated by radical change and budget cuts), as well as current concerns about the role of GPs and their willingness or capacity to mainstream action on health inequalities in the absence of the expertise, networks or policy incentives to do so.

Other aspects of the restructuring, in particular the proposal to realign funding formulas based solely on the age of the population, rather than on area deprivation, will also make reducing health inequalities in the new environment increasingly difficult [31]. Coupled with the clustering of deprivation in certain CCG regions [30], clinical commissioners will not only have to be aware of the scale of particular inequalities in their region, but will also have to actively fight to get resources to address them.

Clinical commissioning certainly has some way to go to dispel the concerns of GPs themselves, and others, in this regard. But it is also true that some of the opportunities recognised by respondents in the present study occur alongside a fresh opportunity to re-engage with health inequalities within the commissioning agenda. Local Authorities will have renewed responsibilities in this new landscape, with a ring-fenced public health budget and central position to tackle the wider determinants of health inequalities. However, there are concerns about the impact on health inequalities of dividing responsibility for clinical and public health commissioning. Clearly, the ability of stakeholders to work together and commit sufficient resources to have a meaningful impact on reducing inequalities within this new arrangement still remains to be seen.

It is worth noting that this study was conducted during a time of great flux for many participants. In particular, many interviews pre-dated the announcement that clinicians other than GPs and wider stakeholders will be involved in CCGs, and most of our respondents were reflecting specifically on GP-only commissioning. Nevertheless, GPs will make up most of the membership of these new commissioning groups, and undoubtedly these reflections on inequalities will be pertinent to the new structures. It is also important to note that local interviewees largely offered opinions based on their experience in particular geographical and clinical areas, and the generalisability of the findings presented here must necessarily be treated with caution given the great variety of approaches to tackling inequalities that have occurred across the country.

Notwithstanding these limitations, this study is a first step in drawing out the significance of the new commissioning arrangements for the goal of reducing health inequalities. Much remains to be seen as to how this will progress as the new structures develop. Nevertheless, our findings suggest that the individual agency of GPs committed to reducing health inequalities, and the ability of CCGs to develop effective partnerships with relevant interest groups, will be key factors in determining whether clinician-led commissioning emerges as an effective mechanism for addressing health inequalities.

## Consent

Written informed consent was obtained from the participant for publication of this report and any accompanying images.

## Competing interests

The authors declare that they have no competing interests.

## Authors’ contributions

SS and GM conceived and designed the project. DT drafted the paper, and co-ordinated comments and contributions. SS, GM and GE made substantive contributions to the background and introduction. All authors except GE conducted interviews, and all authors took part in analysis workshops and attended meetings to shape the direction of study. All authors read and approved the final manuscript.

## Pre-publication history

The pre-publication history for this paper can be accessed here:

http://www.biomedcentral.com/1471-2458/13/274/prepub
